# CHANGES IN DEPRESSIVE SYMPTOMS IN EAST ASIA: A COORDINATED ANALYSIS OF THREE LONGITUDINAL STUDIES

**DOI:** 10.1093/geroni/igz038.987

**Published:** 2019-11-08

**Authors:** Takeshi Nakagawa, Jinmyoung Cho, Dannii Yeung

**Affiliations:** 1 National Center for Geriatrics and Gerontology, Aichi, Japan; 2 Baylor Scott & White Health, Temple, Texas, United States; 3 City University of Hong Kong, Hong Kong, Hong Kong

## Abstract

Evidence for changes in depressive symptoms is relatively sparse in Asian populations. We examined changes in depressive symptoms in China, Korea, and Japan. Data were derived from three longitudinal studies with three measurement waves: the China Health and Retirement Longitudinal Study (CHARLS between 2011—2015), the Korean Longitudinal Study of Aging (KLoSA between 2006—2010), and the Japanese Study of Aging and Retirement (JSTAR between 2007—2011). Participants aged 50—75 years were included in the analysis (CHARLS: n = 10,385; KLoSA: n = 6,683; JSTAR: n = 3,004). Multilevel analyses were conducted separately for each country to examine trajectories of depressive symptoms, controlling for age, age squared, gender, education, marital status, activities of daily living, and morbidity as covariates. Depressive symptoms were measured by the 10-item CES—D. The CES—D score was scaled to a T score metric (M = 50, SD =10) using the score at wave 1 in each country as a reference. Trends in depressive symptoms varied across countries, with stability in China but increase in Korea and Japan (Estimate = —0.05, 0.69, 0.40, respectively). Older Koreans reported higher levels of depressive symptoms than younger adults, whereas a reverse pattern was shown in China. Age differences were not found in Japan. Higher levels of education were associated with lower levels of depressive symptoms in China and Korea, whereas the opposite association emerged in Japan. These country differences will be discussed through the lens of societal and economic factors (e.g., welfare systems and economic recession).

